# Reduced lignin content and altered lignin composition in the warm season forage grass *Paspalum dilatatum* by down-regulation of a *Cinnamoyl CoA Reductase Gene*

**DOI:** 10.1007/s11248-014-9784-1

**Published:** 2014-02-07

**Authors:** Andrea Giordano, Zhiqian Liu, Stephen N. Panter, Adam M. Dimech, Yongjin Shang, Hewage Wijesinghe, Karen Fulgueras, Yidong Ran, Aidyn Mouradov, Simone Rochfort, Nicola J. Patron, German C. Spangenberg

**Affiliations:** 1 Department of Environment and Primary Industries, AgriBio Centre for AgriBioscience, 5 Ring Road, Bundoora, VIC 3083 Australia; 2 La Trobe University, Kingsbury Drive, Bundoora, VIC 3086 Australia; 3 Present Address: Plant Biology Department, Federal University of Viçosa, Av. PH Rolfs s/n, Viçosa, MG Brazil; 4 Present Address: School of Applied Sciences, RMIT University, Plenty Road, Bundoora, VIC 3083 Australia; 5 Present Address: The Sainsbury Laboratory, Norwich Research Park, Norwich, NR4 7UH UK

**Keywords:** C4 forage grass, Cinnamoyl-CoA reductase, Lignin, *Paspalum dilatatum*

## Abstract

C4 grasses are favoured as forage crops in warm, humid climates. The use of C4 grasses in pastures is expected to increase because the tropical belt is widening due to global climate change. While the forage quality of *Paspalum dilatatum* (dallisgrass) is higher than that of other C4 forage grass species, digestibility of warm-season grasses is, in general, poor compared with most temperate grasses. The presence of thick-walled parenchyma bundle-sheath cells around the vascular bundles found in the C4 forage grasses are associated with the deposition of lignin polymers in cell walls. High lignin content correlates negatively with digestibility, which is further reduced by a high ratio of syringyl (S) to guaiacyl (G) lignin subunits. Cinnamoyl-CoA reductase (CCR) catalyses the conversion of cinnamoyl CoA to cinnemaldehyde in the monolignol biosynthetic pathway and is considered to be the first step in the lignin-specific branch of the phenylpropanoid pathway. We have isolated three putative *CCR1* cDNAs from *P. dilatatum* and demonstrated that their spatio-temporal expression pattern correlates with the developmental profile of lignin deposition. Further, transgenic *P. dilatatum* plants were produced in which a sense-suppression gene cassette, delivered free of vector backbone and integrated separately to the selectable marker, reduced *CCR1* transcript levels. This resulted in the reduction of lignin, largely attributable to a decrease in G lignin.

**Electronic supplementary material:**

The online version of this article (doi:10.1007/s11248-014-9784-1) contains supplementary material, which is available to authorized users.

## Introduction

Dallisgrass (*Paspalum dilatatum* Poir.) is a C4 grass native to South America, but widely grown for forage in tropical, subtropical and warm temperate regions of the world. Several polyploid biotypes, including sexual tetraploids, apomictic pentaploids and hexaploids have been described (Casa et al. 2002). Valued for its vigour, dallisgrass produces yields of up to 15 tons of dry matter per hectare (Cook et al. 2005) with crude protein concentrations of up to 18.6 % (Baréa et al. 2007). It can withstand heavy grazing and is somewhat tolerant to frost and water stress (Hutton and Nelson 1968; Robinson et al. 1988).

With the widening of the tropical belt due to global climate change (for review see Seidel et al. (2008) the market for C4 forages grasses is set to increase. However, while the forage quality of *P. dilatatum* is higher than that of other C4 forage grass species, digestibility of warm-season grasses is, in general, poor compared with most temperate grasses (Wilson 1994). This is due to the presence of lignin deposits in the thick-walled parenchyma bundle-sheaths around each vascular bundle (Wilson 1993). Lignin acts as a physical barrier to the microbial enzymes that digest polysaccharides, limiting the achievable degradation of cellulose and hemicellulose and limiting the digestible energy available to ruminants (Jung and Allen 1995). Consequently, modification of the lignin biosynthetic pathway, either by analyses of mutants or by genetic modification, is of great interest (Gordon and Neudoerffer 1973; Barrière et al. 1994; Halpin et al. 1994; Bernard-Vailh et al. 1996; Guo et al. 2001; Chen et al. 2004; Li et al. 2008; Hisano et al. 2009; Tu et al. 2010).

Lignin, found in the cell walls of vascular plants, is a complex heteropolymer resulting from radical coupling reactions of three main monolignols: *p*-coumaryl alcohol, coniferyl alcohol and sinapyl alcohol. The degree of methoxylation of the phenyl ring differs in each subunit giving rise to hydroxyphenyl (H), guaiacyl (G) and syringyl (S) lignin, respectively (Rogers and Campbell 2004).

The first step in the lignin-specific branch of the phenylpropanoid pathway is catalysed by cinnamoyl-CoA reductase (CCR). This enzyme regulates the carbon flux towards lignin and is thus a viable target to alter lignin levels (Lacombe et al. 1997; Baucher et al. 1998). Genes encoding CCR have been studied in many species including the model plants *Arabidopsis thaliana*, *Oryza sativa* and *Populus tremuloides* (Costa et al. 2003; Li et al. 2003; Kawasaki et al. 2006). *CCR1* genes have been found to be preferentially expressed in the stems of *Panicum virgatum*, *A. thaliana*, *Medicago truncatula*, *Triticum aestivum*, *Lolium perenne* and *Zea mays* (Pichon et al. 1998; Lauvergeat et al. 2001; Goujon et al. 2003; Larsen 2004; Ma and Tian 2005; Escamilla-Treviño et al. 2010; Tu et al. 2010; Zhou et al. 2010) and are thus thought to be involved in lignification. A reduction in the total lignin level and changes in monolignol composition were positively correlated with improved digestibility in the brown-midrib (*bm*) mutants of *Z. mays* as well as in transgenic plants in which caffeic acid 3-*O*-methyltransferase (COMT) was down-regulated (Baucher et al. 1998; Casler and Vogel 1999; Piquemal et al. 2002; Chen et al. 2004; Hisano et al. 2009; Tu et al. 2010; Tamasloukht et al. 2011). Down-regulation of *CCR1* expression has been achieved in *Nicotiana tabacum* (Dauwe et al. 2007), *Medicago sativa* (Jackson et al. 2008), *Solanum lycopersicon* (Van der Rest et al. 2006) *Z. mays* (Park et al. 2012) and hybrid poplar (*Populus tremula* × *Populus alba*) (Leplé et al. 2007) as well as in the C3 forage, *L. perenne*, in which a decrease in total lignin was observed to correlate with improved digestibility (Tu et al. 2010).

Even moderate increases in digestibility have significant economic consequences; in *L. perenne* a 5–6 % increase in digestibility was estimated to increase summer milk production in southern Australia by up to 27 % (Smith et al. 1998). In this study we have isolated and characterised putative *CCR1* cDNAs from *P. dilatatum* cv. Primo, a tetraploid sexually reproductive commercial cultivar. We describe the pattern of lignin deposition and *CCR1* transcript levels during plant development and analyse the impact of down-regulating *CCR1* on lignin composition and digestibility. Gene silencing was triggered by expression of a frame-shift mutant of a *CCR* gene, which was delivered separately to the selectable marker cassette. Both cassettes were delivered free of vector backbone.

## Experimental procedures

### Plant material

Seeds of *P. dilatatum* cv. Primo, a tetraploid (2*n* = 4*x* = 40) sexually reproductive cultivar, kindly provided by Gustavo Schrauf, Facultad de Agronomía de la Universidad de Buenos Aires (FAUBA), were grown in glasshouses (21 °C, 14 h photoperiod/14 °C, 10 h dark period) or growth cabinets (21 °C, 14 h photoperiod/16 °C, 10 h dark period).

### Isolation of putative cinnamoyl-CoA cDNAs from *P. dilatatum*

Reference sequences of *CCR1* genes from *Sorghum bicolor*, *Z. mays*, *P. virgatum* and *L. perenne* were aligned and the consensus sequence was used to design primers to conserved regions. Sequences of all primers are provided as supporting information (Online Resource 1). Total RNA was extracted from 100 mg of ground tissue (stems, roots, leaf blades and inflorescences) from the final reproductive stage of *P. dilatatum* cv. Primo plants using the RNeasy^**®**^ Plant Mini Kit (Qiagen, Hilden, Germany) according to manufacturer’s instructions. A cDNA library was prepared from pooled RNA using the SMART™ cDNA Synthesis kit (Clontech Laboratories, Mountain View, CA, USA). First strand cDNA was reverse transcribed using SMARTScribe™ Reverse Transcriptase and SMART IV™ oligonucleotides (Clontech Laboratories) and putative *CCR* genes were amplified from this template. Further transcripts were identified using the *SMARTer*™ RACE cDNA Amplification Kit (Clontech Laboratories). After re-amplification with proofreading polymerases, full-length coding sequences were cloned and sequenced using ABI Sanger Sequencing and Big Dye Terminator v3.1 (Life Technologies Corporation, Carlsbad, USA).

### Southern hybridisation analysis

Genomic DNA was isolated from leaf tissue using a standard cetyltrimethylammonium bromide protocol (Doyle and Doyle 1987) and 10 μg was digested with one or more of the restriction enzymes *Hin*dIII, *Eco*RI and *Sac*I in separate reactions and separated on a 0.8 % (w/v) agarose gel. Following electrophoresis, DNA was transferred to a Hybond N membrane (GE Healthcare, Little Chalfont, UK) using established protocols (Sambrook et al. 1989). A *PdCCR*-specific probe was generated using a PCR-based digoxigenin (DIG) Probe Synthesis Kit (Roche, Basel, Switzerland) according to the manufacturer’s instructions. Probes specific to the promoter of the polyubiquitin gene from *Z. mays*, present in the CCR-frameshift transgene cassette, and to the *npt*II selection gene were also made. Sequences of all primers are provided in Online Resource 1. Hybridisation with the Pd*CCR*-specific probe was performed at 58 °C overnight and with the *Z. mays* polyubiquitin gene promoter and *npt*II gene probes at 55 °C overnight. A chemiluminescent detection protocol was used as per manufacturer’s instructions (DIG Luminescent Detection Kit, Roche).

### Phylogenetic analysis

The deduced amino acid sequences of the *PdCCR* transcripts identified in this study were aligned with previously derived *CCR* gene sequences as well as with sequences identified from publically available complete plant genomes using BLASTx and tBLASTn (cut-off of e^−4^). Reciprocal BLAST was performed against the *A. thaliana* and *O. sativa* genomes. Alignments were made using ClustalX (Larkin et al. 2007) and imported into Mesquite (Maddison and Maddison 2011) for refinement. A preliminary phylogenetic analysis showed that the clade containing the conserved NWYCY motif was monophyletic. Sequences from this clade were then subjected to rigorous analyses using the two most-closely related genes encoded in the genome of the bryophyte, *Physcomitrella patens* (from which no genes containing the conserved motif were identified) as an out-group. Ambiguously aligned characters were excluded in Mesquite, resulting in a matrix of 54 taxa and 313 characters. A maximum likelihood phylogeny was inferred using PhyML v3.0 (Guindon and Gascuel 2003) with the LG substitution model and eight categories of substitution rates. The alpha value and number of invariable sites were calculated from the data sets. Branch support was assessed using ML bootstrap analysis (PhyML with eight categories and 100 replications).

### Expression analysis

RNA was purified from plant tissue using the ZR Plant RNA MiniPrep™ Kit (Zymo Research, Irvine, CA) or the RNeasy Plant Mini Kit (Qiagen) according to manufacturer’s instructions. Expression of *PdCCR* during development of non-transgenic plants was analysed by quantitative reverse-transcriptase PCR (qRT-PCR) designed to amplify all *CCR* transcripts identified. Data was normalised using expression values of elongation factor-1 alpha (*EF1α*) as a reference gene (Silveira et al. 2009). Quantification was performed using SYBR Green Mastermix (Roche, Basel, Switzerland) with 200 nM of each primer for *PdCCR* amplification and with 300 nM of each primer for *EF1α* expression analysis. Cycling conditions were as follows: 95 °C for 10 min, 40 cycles of 95 °C for 30 s, and 60 °C (*EF1α*) or 63 °C (*PdCCR*) for 1 min, and 1 cycle of 95 °C for 1 min, 60 °C for 30 s and 95 °C for 30 s. Expression of *PdCCR* in transgenic plants was determined in the same way except that a set of primers spanning the coding and 3’ untranslated region was designed to amplify all *CCR* transcripts but to exclude amplification of transcript from the transgene cassette. Cycling conditions for this assay were as follows: 95 °C for 10 min, 40 cycles of 95 °C for 30 s, and 60 °C (*EF1α*) or 68 °C (*PdCCR*) for 1 min, and a hold of 95 °C for 1 min followed by a dissociation curve (60 °C to 95 °C). Sequences of all primers are provided in Online Resource 1. A tenfold dilution series of standard templates (linear double-stranded DNA) was prepared for absolute quantification. Standard error was calculated from three biological replicates. Amplification was performed on either an Mx3005P (Stratagene, Santa Clara, CA, USA) or a C1000 thermal cycler with the CFX96 real-time PCR detection system (Bio-Rad, Hercules, CA, USA). Results were analysed using the MxPro (Stratagene) or CFX Manager (Bio-Rad) software packages. Relative quantification was calculated according to the 2^−∆∆Ct^ method (Livak and Schmittgen 2001).

### Construction of expression cassettes

Expression cassettes consisting of the promoter and 5′ untranslated region (UTR) of the polyubiquitin (*Ubi*) gene from *Z. mays* (Toki et al. 1992), followed by the entire coding sequence of *PdCCR1*-*1* with a frame-shift created by deleting the seventh base-pair downstream of the start codon and the 3′ UTR comprising transcriptional terminator and polyadenylation site from the fructosyltransferase 4 (*FT4*) gene from *L. perenne* were synthesised (GeneArt, Life Technologies, Regensberg, Germany). A plant selectable marker cassette consisting of the promoter and 5′ UTR from the actin (*act1*) gene from *O. sativa* (McElroy et al. 1990) followed by the neomycin phosphotransferase (*npt2*) gene was also synthesised. This cassette was terminated with the 3′ UTR, comprising transcriptional terminator and polyadenylation site, from the octopine synthase (*oct*) gene from *Agrobacterium tumefaciens*. Cassettes were liberated from the vector backbones by restriction digestion and separated from the plasmid DNA fragment using the Elutrap system (Whatman, Maidstone, UK) according to the manufacturer’s instructions.

### Generation of transgenic *P. dilatatum* plants

A single genotype, Primo-11, was selected on the basis of observing shoot regeneration from embryogenic callus (EC) derived from mature seeds of *P. dilatatum* cv. Primo. This approach, including methods for surface sterilisation, callus induction from mature seed and vegetative in vitro tillers, shoot regeneration and maintenance of vegetative in vitro tillers, was adapted from Bajaj et al. (2006). Modifications to this method include a 15 min immersion of seeds in 50 % (v/v) sulphuric acid, the omission of endophyte control treatments, Murashige and Skoog (MS) media (Murashige and Skoog 1962) solidified with 0.7 % (w/v) agar (Sigma, St. Louis, MO, USA), the use of MS supplemented with 3 % (w/v) sucrose and 1 μM Kinetin (MSK) for shoot regeneration, the use of MS supplemented with 3 % (w/v) sucrose and 15 μM 6-benzylaminopurine (BA, Sigma, St. Louis, MO, USA) for maintenance medium and the use of MS supplemented with 3 % (w/v) sucrose and 22.6 μM 2,4-dichlorophenoxyacetic acid (2,4-D, Sigma, St. Louis, MO, USA) for callus induction medium.

Five to seven days prior to transformation EC were preconditioned by osmotic treatment on MS medium supplemented with 3 % sucrose, 64 g/L mannitol and 6 μM 2,4-D and incubated in the dark for 4 h at 24 ± 2 °C. EC were then bombarded with 0.6 μm gold particles coated with equal quantities of cassette DNA using the PDS 1000/He system (Bio-Rad) with a system pressure of 1,100 psi and a vacuum degree of 9.5 kPa (28 inches of mercury). Bombarded EC were kept in the dark for 16 h at 24 ± 2 °C before transfer to callus induction medium supplemented with 50 mg/L paromomycin as the selective agent. After 1 week, EC were transferred to regeneration medium supplemented with 50 mg/L paromomycin and incubated for 2 weeks at 24 ± 2 °C under a 16 h photoperiod at a photon flux density of 75 μM m^−2^ s^−1^. EC were subcultured every 2 weeks for 6 weeks on regeneration medium supplemented with 50 mg/L paromomycin. Putative transgenic plants with established roots were transferred to potting mix and grown in the glasshouse in conditions described above. Transgene was confirmed using qPCR to detect the endogenous gene EF1α, the selectable marker cassette and the *PdCCR* frameshift cassette. Sequences of all primers are provided as supporting information (Online Resource 1). Transgenic status was verified and copy number was determined by Southern hybridisation analysis as described above. Each transgenic line and two non-transgenic lines of the same genetic background, also regenerated in tissue-culture, were cloned into three biological replicates. Tissue samples of leaf blades and pseudostems of equivalent maturity were taken from each clone. Each sample was frozen in liquid nitrogen, ground and divided to provide material for expression and biochemical analyses. Each sample was analysed in triplicate to provide technical replicates.

### Histochemical staining of lignin

The Mäule reagent specifically stains syringyl (S) lignin subunits red and guaiacyl (G) subunits brown (Iiyama and Wallis 1990; Lin and Dence 1992). Transverse sections of stems at different development stages were stained as previously described (Chen et al. 2002). Images were captured using a Leica MZ FLIII stereo fluorescence microscope with a Leica DFC300F colour camera (Leica Microsystems, Wetzlar, Germany).

### Lignin content and composition analysis

Tissue samples were freeze-dried and homogenised to a fine powder using a mixer mill (MM400, Retsch Technology, Haan, Germany) at 25 Hz for two minutes. For isolation of cell walls, 100 mg of powdered leaf blade or stem tissue was sequentially extracted with hot water, ethanol and acetone. The remaining cell wall extract was used for determination of total lignin content and subunit composition analysis. The lignin content of leaf and stem tissues was quantified using the acetyl bromide soluble lignin method (Iiyama and Wallis 1990) with 6 mg of cell wall extract used per assay. Monolignol composition was determined with the thioacidolysis method (Rolando et al. 1992) using 8 mg of each cell wall extract per assay. Statistical analysis (Student *t* Test, *P* < 0.05) was carried out using Excel 2003 (Microsoft, Seattle, WA, USA).

### Metabolite analysis of transgenic *Paspalum dilatatum* plants relative to control plants

Tissue samples were freeze-dried and homogenised to a fine powder. For the analysis of intermediate metabolites involved in monolignol synthesis, 50 mg of powdered leaf-blade tissue was extracted twice with one mL of 80 % methanol. The supernatants were combined and used directly for liquid chromatography-mass spectrometry (LC–MS) analysis. High-performance liquid chromatography (HPLC) separation was achieved using a 150 × 2.1 mm Agilent Eclipse XDB 1.9 μm C18 column fitted to an Agilent 1290 Infinity HPLC system (Agilent Technologies, Santa Clara, CA, USA). Metabolites were eluted from the column using a gradient mobile phase (containing water and acetonitrile) at a flow rate of 0.3 mL/min. The compounds were detected with a LTQ Orbitrap Velos mass spectrometer (Thermo Fisher Scientific, Waltham, MA, USA) with a heated electrospray ionisation source and a positive–negative switching scanning mode (both over the 80–2,000 m/z range). Standard compounds, namely *p*-coumaric acid, phenylalanine, ferulic acid, sinapic acid, and caffeic acid (all from Sigma-Aldrich) were analysed under the same conditions to identify metabolites of interest within the samples. Quantification of metabolites was performed using LCquan 2.0 software (Thermo Fisher Scientific) and statistical analysis (Student *t* Test, *P* < 0.05) was carried out using Excel 2003 (Microsoft).

### Near-infrared reflectance spectroscopy (NIRS)

Vegetative herbage samples (cut 50 mm above the soil surface) were dried for 72 h at 55 °C and ground to a fine powder using a MM400 mixer mill (Retsch Technology) at 30 Hz for 2 min. Spectral data analysis was obtained and analysed and nutritive value estimated as described by Tu et al. (2010).

## Results

### Isolation and characterisation of *PdCCR1*

**Fig. 1:**
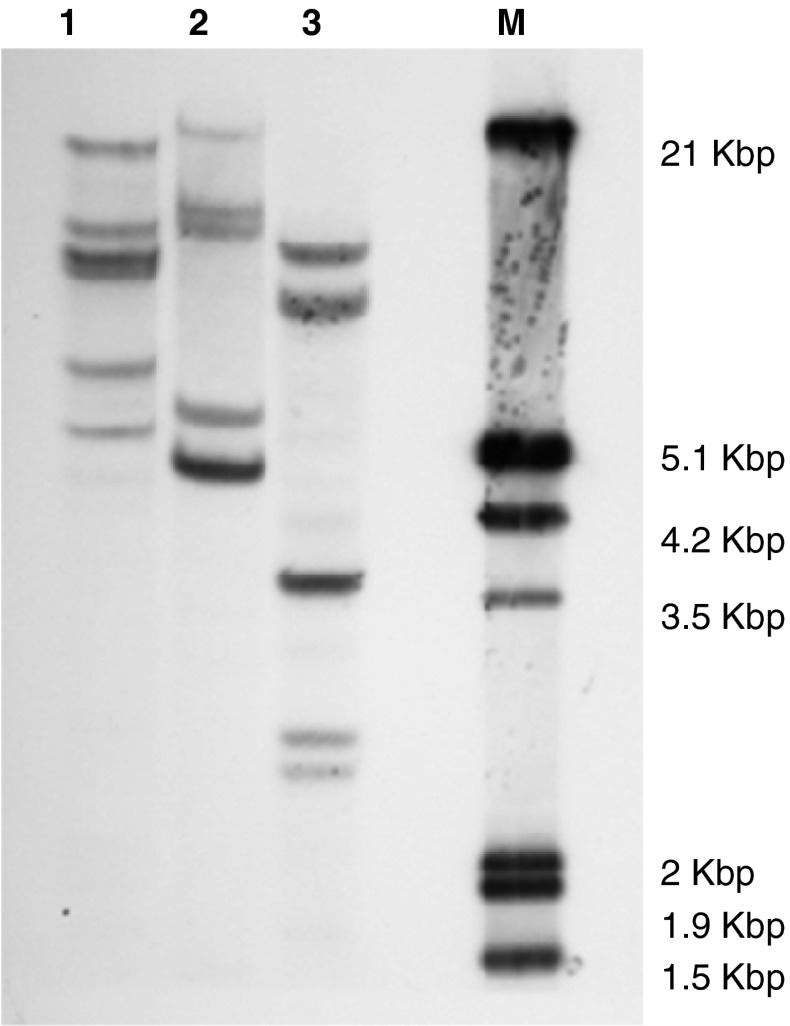
Southern hybridisation analysis of genomic DNA (10 μg per *lane*) purified from *P. dilatatum* plants digested with *Hin*dIII (1), *Eco*RI (2), *Sac*I (3) and probed with a 337 bp DIG-labelled fragment of a *CCR* gene amplified from *P. dilatatum*. *M* DIG Marker III

Transcripts encoding open reading frames, from which the inferred translations suggested functional CCR proteins, were isolated by use of primers designed to conserved regions (NPDDPK and DYDAI; RV/MVFTS and EQMVEP; VVNPVL and TVNASI) of the coding sequences of *CCR* genes from monocotyledonous species and rapid amplification of cDNA ends (RACE). Three cDNAs with high identity (97–99 %) in the coding region but divergent in the 3′ UTRs were identified. Southern hybridisation analysis with a probe from the coding region revealed four clear bands and one doublet band in DNA digested with *Hin*dIII and five bands, one having twice the intensity of the others, can be seen in DNA digested with *Eco*RI and *Sac*I (Fig. 1). The simplest interpretation is that there are three loci on each of the two sub-genomes although it possible that the bands are different alleles of fewer loci.

**Fig. 2:**
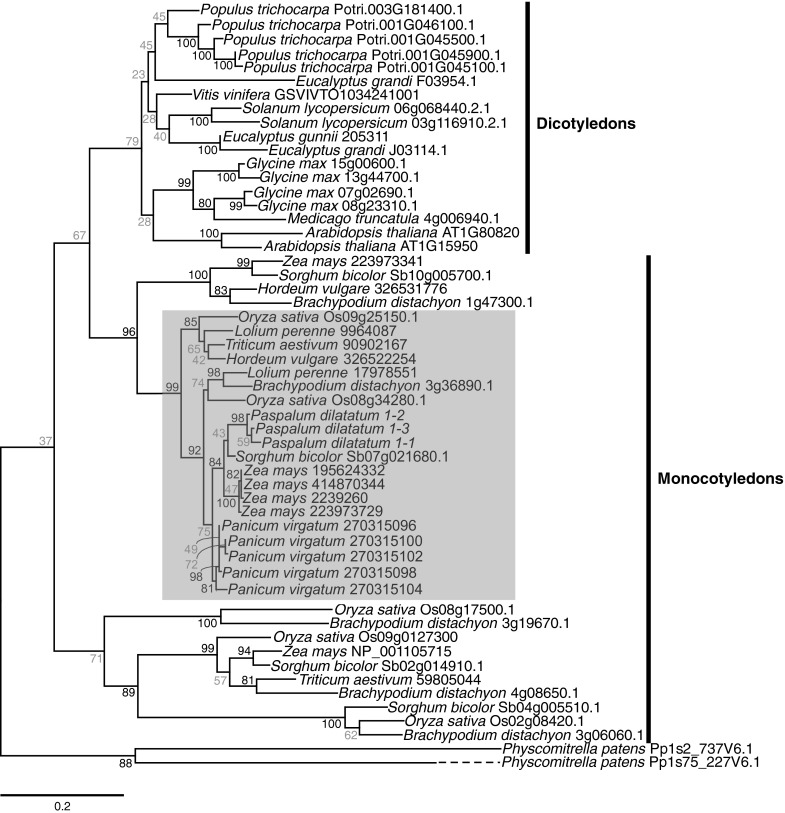
Maximum likelihood phylogeny of deduced CCR protein sequences from Viridaeplantae. *Numbers* at nodes correspond to ML bootstrap support. Moderate to strong support (>80) is shown in *black*, and weak support (>79) is shown in *grey*. The *shaded box* highlights CCR1 proteins from monocotyledons. The *dashed line* indicates a long branch

The inferred amino acid sequences of the coding regions from the three full-length cDNAs isolated were compared to previously characterised CCR proteins. The motif NWYCY, previously suggested as the catalytic site for the enzymatic activity, was present in all cloned cDNAs. A βαβ secondary structure at the N-terminus, which corresponds to the conserved binding fold domain NAD/NADP(H)-dependent dehydrogenases and reductases, was located between residues 22 and 51 of the predicted proteins encoded in the cDNAs amplified from *P. dilatatum*. Phylogenetic analysis of CCR proteins encoded in plant genomes revealed three major clades: one comprised of dicotyledons and two comprised of monocotyledons, each with moderate support (Fig. 2). A separation that would support the functional divergence of CCR in higher plants into those with roles in defense-related lignin deposition and others involved in developmentally related lignification was not observed. In dicotyledons, the proteins associated with each function, for example CCR1 and CCR2 from *A. thaliana*, were shown to be the result of duplications that occurred post-speciation (Fig. 2). The sequences from *P. dilatatum* grouped with strong (99 bootstraps) support with proteins from monocotyledons described as CCR1, including those from *L. perenne*, *T aestivum* and *Z. mays* (accession numbers 17978551, 90902167 and 2239260, respectively), for which function has been linked to lignin deposition. Within this clade, the *P. dilatatum* proteins are closely related to proteins encoded in the genomes of other C4 species (*P. virgatum*, *Sorghum bicolor* and *Z. mays*) (Fig. 2). We have therefore named the three coding sequences cloned from *P. dilatatum* as *PdCCR1*-*1*, *PdCCR1*-*2* and *PdCCR1*-*3* (KC886283-5).

### The spatio-temporal pattern of *PdCCR1* expression during plant development correlates with lignification

Three developmental stages of *P. dilatatum* cv. Primo, vegetative (V), early reproductive (R1) and late reproductive (R2), were selected for analysis of *PdCCR1* expression, lignin deposition and composition. At V stage true stems have not yet formed and the plant is described as juvenile. Pseudostems are composed of the folded or rolled bases of leaves that have yet to emerge within a sheath. The reproductive stage is divided into early reproductive, R1, defined by the emergence of an inflorescence and late reproductive, R2, when the inflorescence is fully emerged and anthesis has commenced. At R1 and R2 stages almost all stems contain three internodes, basal (I1), middle (I2) and upper (I3), into which tissue was divided for analysis.

The spatio-temporal expression pattern of *PdCCR1* expression through plant development was determined by qRT-PCR analysis of leaf blades and pseudostems sampled from the V stage and in the stem internodes and corresponding leaf from each internode at the R1 and R2 developmental stages using primers that detected all of the transcripts identified. The content of lignin as percentage of cell wall residue from the same tissues was quantified using an acetyl bromide soluble lignin assay.

**Fig. 3:**
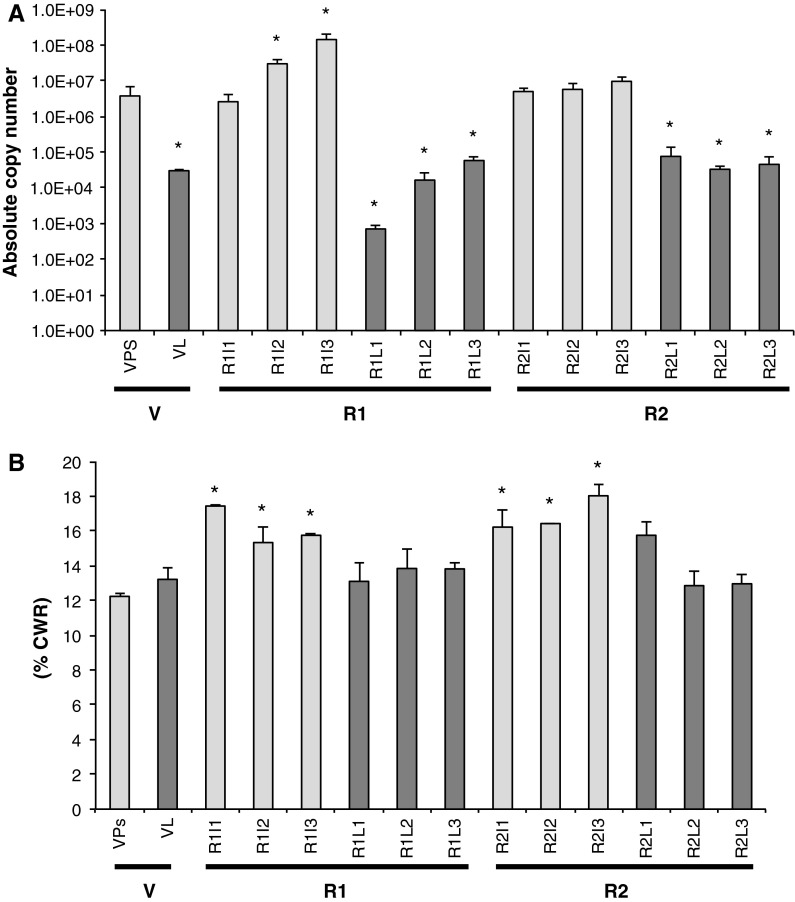
**a** *PdCCR1* expression and **b** lignin content of cell wall extracts as percentage of cell wall residue (% CWR) quantified using an acetyl bromide soluble lignin assay in *P. dilatatum* tissues through development. Values are average and standard errors of three biological replicates. *V* Vegetative stage (*Ps* Pseudostem, *L* leaf), *R1* early reproductive stage, *R2* late reproductive stage (*I1* internode 1, *I2* internode 2, *I3* internode 3, *L1* leaf 1, *L2* leaf 2, *L3* leaf 3). *Asterisks* indicate a significant difference (*t* test) relative to V stage pseudostems (*P* < 0.05)

**Fig. 4:**
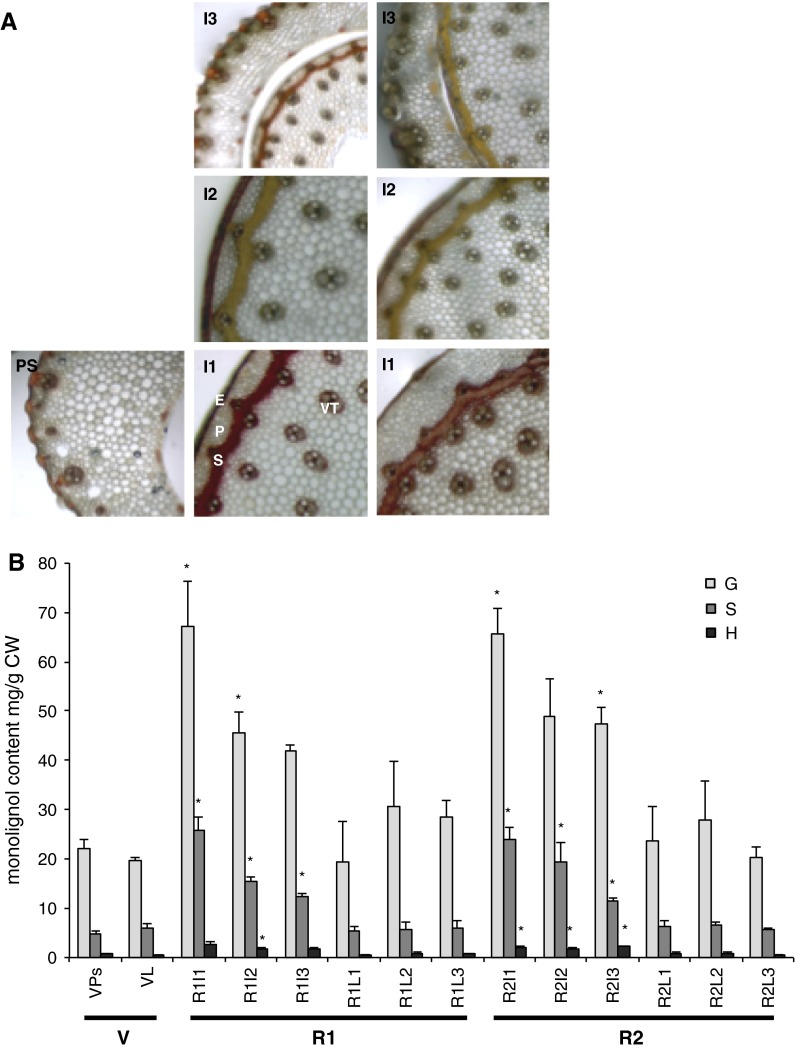
Composition of lignin in *P. dilatatum* stems and leaf blades through development as determined by **a** Mäule staining of transverse sections and **b** thioacidolysis of cell wall extracts. *Asterisks* indicate a significant difference (*t* test) relative to V stage pseudostems (*P* < 0.05). *V* Vegetative stage (*Ps* Pseudostem, *L* leaf), *R1* early reproductive stage, *R2* late reproductive stage (*I1* internode 1, *I2* internode 2, *I3* internode 3, *L1* leaf 1, *L2* leaf 2, *L3* leaf 3). Epidermal cells (*E*), Parenchyma cells (*P*), Sclerenchyma cells (*S*), Vascular tissue (*VT*), *scale bar* = 100 μm. mg/g CW = mg per gram of dry cell wall residues (mean and standard error of three biological replicates) *G* guaiacyl lignin, *H* hydroxyphenyl lignin, *S* syringyl lignin

*PdCCR1* transcripts were detected in leaf blades and pseudostem tissues sampled from plants at the V stage and also in all internodes of the stem and in all leaf blades from plants at both reproductive stages. The highest levels of expression were observed in tissues sampled from the uppermost internodes of stems at the early reproductive stage (R1I3) (Fig. 3A). There was a correlation between lignin deposition and the development of true stems. Significantly more lignin was found in all R1 and R2 stage internodes than in V stage pseudostems (Fig. 3B). Lower levels of *PdCCR1* transcripts were observed in tissues in which lignification was minimal but also in highly lignified tissues such as stems at R2 stage, the latter of which were the most lignified tissues analysed (Fig. 3). There was no significant difference in the lignin content of pseudostems and leaf blades sampled at the vegetative stage although *PdCCR1* expression in leaf blades was significantly lower than in pseudostems (Fig. 3). This indicates that lignin deposition occurs early in V stage leaf development and that expression of *PdCCR1* declines after the emergence of the blade. To determine lignin composition, transverse sections of stems were taken from all three internodes at the R1 and R2 stages and also from pseudostems sampled at the V stage. Sections were stained histochemically using Mäule reagent, which stains G and S lignin brown and red, respectively. Lignin accumulation was observed in vascular, epidermal and sclerenchyma cells. The transition from vegetative to reproductive development was associated with an increase in the number of Mäule-stained cells and a colour shift from brownish-yellow to red indicating an increase in S lignin as the plant matures (Fig. 4A). Within the R1 and R2 stages an increasing amount of red coloration was observed in sections from the basal parts of the stems compared to the less-developed upper internodes (Fig. 4A).

Thioacidolysis of cell wall extracts to determine lignin subunit composition also showed that the content of G- and S-lignin increased as plants transitioned to the reproductive stage (Fig. 4B). In particular, the basal internodes of stems at the early reproductive stage (R1I1) contained five times as much S-lignin and twice as much G-lignin as pseudostems at V stage. This resulted in a change in S/G ratio from 0.19 to 0.35. Within reproductive stems the content of G- and S-lignin decreased with height. The S/G ratio was greatest in basal internodes (0.35 at R1 stage and 0.33 at R2 stage) and lowest in uppermost internodes (0.27 at R1 stage and 0.23 at R2 stage) (Fig. 4B). Conversely, no significant difference was observed in the lignin composition of leaf blades with the S/G ratio remaining between 0.20 and 0.26 in samples taken at all stages of development (Fig. 4B). The content of H-lignin remained low, typically 2–3 % of total lignin, in all tissues sampled (Fig. 4B).

### Downregulation of *PdCCR* in transgenic *P. dilatatum* plants

Putative transgenic *P. dilatatum* plants, resistant to paromomycin were initially tested for the presence of transgenes by Southern hybridisation analysis (see Online Resource 2). Probes specific to the promoter of the *polyubiquitin* gene from *Z. mays*, designed to detect the *PdCCR* cassette, and to the *nptII* gene indicated that >5, 2 and 5 copies of the *PdCCR* cassette and >5, 1 and 6 copies of the *nptII* cassette were integrated in lines 76, 78 and 87 respectively (see Online Resource 2). In each line, the pattern of bands obtained with the two probes differed, indicating separate integrations of the *PdCCR* and *nptII* cassettes (see Online Resource 2), which suggests that the selectable marker may be removed by segregation in subsequent generations.

**Fig. 5:**
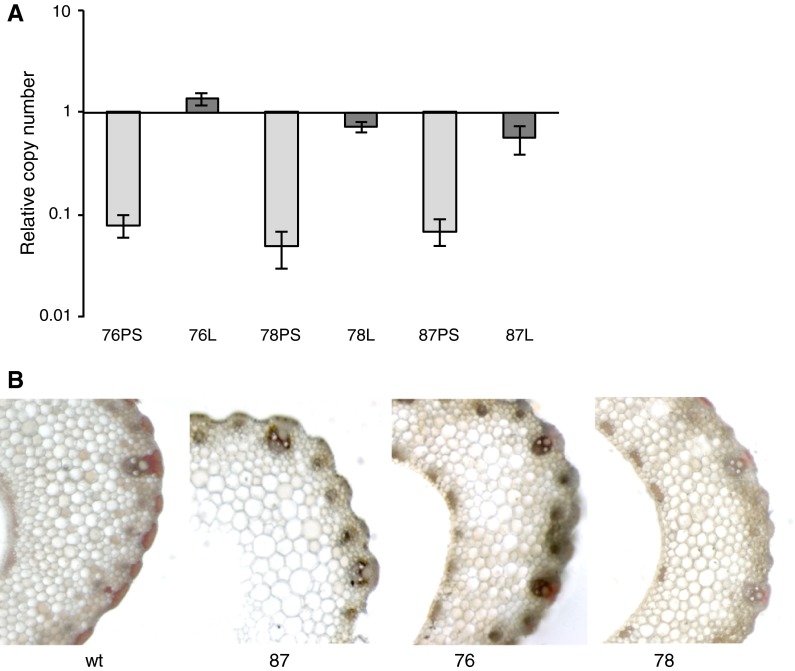
**a** Expression of *CCR1* in pseudostems and leaf blades of transgenic lines of *P. dilatatum* normalised to the wild-type. Data shown is mean and standard error of three replicates of *each line*. *PS* pseudostem, *L* leaf blade **b** Mäule staining of transverse sections of pseudostems from wild-type and transgenic lines of *P. dilatatum*. All samples were taken from plants at the vegetative stage

In all three transgenic lines, the abundance of *PdCCR* transcripts in pseudostem tissue was found to be at least ten-fold lower than that of wild-type control plants (Fig. 5) indicating that the frame-shift cassettes had successfully induced gene-silencing. In leaf blades the reduction was more moderate with two lines showing only a two to three fold reduction in *PdCCR* transcript level (Fig. 5). However, expression of *PdCCR1* transcripts in V stage leaf blades declines during development and lignin deposition is likely to have taken place when the young leaf was still rolled and part of the pseudostem (Fig. 4). Therefore, although leaf blades and pseudostems show comparable lignin content (Fig. 3B) and composition (Fig. 4), pseudostem tissue is the most appropriate V-stage tissue in which to compare *PdCCR1* expression levels.

Although lignin content is lower in the vegetative stage than in reproductive stages,

subsequent analysis of lignin content and composition focussed on leaf blades from plants at the V stage, since this is the typical stage where grazing occurs and where gains in digestibility will be of greatest consequence. Lignin content in leaf blades was reduced by 20 % in line 76 (Table 1). Thioacidolysis analysis detected a decrease in the level of guaiacyl (G) subunits leading to a significant change in the S/G ratio all three lines (Table 1). This reduction in lignin in pseudostems sampled at the V stage was also observed histochemically using Mäule reagent. Transgenic lines were less intensely stained when compared to wild-type controls (Fig. 5B). Additionally, metabolic profiling showed that the hydroxycinnamates *p*-coumaric acid, ferulic acid and caffeic acid were present at significantly higher levels in transgenic lines compared to the wild-type plants, indicating that intermediates of lignin-biosynthesis were re-directed into other pathways (see Online Resource 3).

**Table 1:** Lignin content (% dry cell wall residues), composition (mg/g dry cell wall residues), and S/G ratio in leaf blades of transgenic lines and wild-type controls

	AcBr-soluble Lignin	G Lignin	S Lignin	H Lignin	S/G
Wild-type control	15.07 ± 0.38	6.47 ± 0.79	1.69 ± 0.23	0.11 ± 0.02	0.26
78	13.94 ± 0.31	5.28 ± 0.63	1.70 ± 0.24	0.10 ± 0.02	0.32*
87	15.39 ± 0.55	5.07* ± 0.17	1.57 ± 0.02	0.12 ± 0.00	0.31*
76	12.28* ± 0.59	4.11 ± 0.45	1.63 ± 0.13	0.09 ± 0.01	0.40*

Error bars indicate standard errors (*n* = 3). Asterisks indicate a significant difference (*t* test) relative to the wild-type control (*P* < 0.05)

Estimation of in vivo dry matter digestibility (IVVDMD) was performed using near-infrared reflectance spectroscopy (NIRS) as per Tu et al. (2010). Two transgenic lines showed a significant increase in IVVDMD in leaf blades (up to 4 %) in comparison to wild-type control lines, which correlated with a reduction in lignin (Online Resource 4).

## Discussion

The influence of lignin composition on cell wall digestibility has been studied in a number of forage species (Jung and Vogel 1986; Buxton and Russell 1988; Akin 1989; Jung and Deetz 1993; Grabber 2005). Increases in global mean temperature and expansion of the tropical zone mean that C4 grasses will become suitable forage crops in some regions that have historically been temperate. *P. dilatatum*, which is already grown for forage in sub-tropical regions, is a candidate species. In general, the lignin content (% dry cell wall) of C4 grasses is higher than that of C3 grasses (Jung and Vogel 1986). The lignin content of pseudostems from *P. dilatatum* plants at the early reproductive stage was found to be 5 % higher than that reported for equivalent tissues in the C3 grass *L. perenne* (Tu et al. 2010). Similarly, the basal internodes of stems at the late reproductive stage were found to have 5 % more lignin than equivalent tissue from *F. arundinacea* (Chen et al. 2002). Lignin composition in monocotyledons changes as plants transition from vegetative to reproductive developmental stages with S-lignin increasing and G-lignin decreasing upon flowering (Anterola and Lewis 2002; Chen et al. 2002; Guillaumie et al. 2008; Tu et al. 2010). In *P. dilatatum,* total lignin content increased through development with the percentage of H-lignin remaining constant but that of S- and G-lignin increasing five- and two-fold respectively.

Phylogenetic analysis showed that the putative CCR1 predicted proteins isolated from *P. dilatatum* in this study are closely related to CCR1 proteins involved in lignin deposition from other monocotyledonous plants, including those from wheat and *L. perenne*. The complete genomes of the monocotyledons *Z. mays*, *O. sativa* and *B. distachyon* all encode additional CCR proteins that resolved outside of the clade containing the CCR1 predicted proteins from *P. dilatatum* (Fig. 2). These include a protein annotated as CCR2 from *T. aestivum* (59805044) (Fig. 2). It is therefore likely that the *P. dilatatum* genome encodes CCR proteins additional to those described in this study.

Using primers designed to detect the three full length cDNAs isolated from *P. dilatatum*, it was found that transcripts of *PdCCR1* were most abundant in tissues undergoing active lignification (R1, internodes two and three) and were less abundant in tissues such as R2 stage internodes and V stage leaf blades (Fig. 3) in which lignin had already accumulated. These observations are consistent with the idea that CCR1 is involved in constitutive lignification. This is similar to results reported for other species including *L. perenne* (Tu et al. 2010) and *Leucaena leucocephala* (Sirvastava et al. 2011).

Transgenic technologies have been widely used over the last decade to modify lignin content and composition in various model plants and crop species. Down-regulation of *CCR* in species such as *N. tabacum* (Piquemal et al. 1998; Ralph et al. 1998; Chabannes et al. 2001; O’Connell et al. 2002; Dauwe et al. 2007), *S. lycopersicum* (Van der Rest et al. 2006) *M. sativa* (Jackson et al. 2008), *A. thaliana* (Goujon et al. 2003) and *L. perenne* (Tu et al. 2010), has generally resulted in a reduction in lignin content. A reduction of lignin content, in particular large reductions of more than 30 %, have been associated with phenotypic abnormalities such as a reduction in plant size (Piquemal et al. 1998; Ralph et al. 1998; Chabannes et al. 2001; Goujon et al. 2003; Van der Rest et al. 2006; Dauwe et al. 2007; Prashant et al. 2011), delayed senescence (Mir Derikvand et al. 2008), delayed flowering (Prashant et al. 2011) and retarded seed development (Jones et al. 2001) or compromised pathogen defence (Prashant et al. 2011). These deleterious phenotypes infer that a very large reduction in lignin content is incompatible with normal growth and plant development. This suggests that there may be an optimal level to which lignin can be decreased for enhanced digestibility without an adverse effect on plant growth. *P. dilatatum* plants in which *CCR* was down-regulated showed no obvious deleterious developmental phenotypes compared to non-transgenic control plants when grown in standard glasshouse conditions. A moderate reduction in total lignin content (up to 20 %) was achieved in leaf blades. A decrease in levels of guaiacyl (G) subunits (up to 37 %) was observed in leaf blades, leading to an increase in the S/G ratio since syringyl (S) and hydroxyphenyl (H) subunit levels were not affected. These changes in composition are consistent with those observed in the *ZmCCR1* ^−^ mutant of *Z. mays* which showed only a 10 % reduction in lignin content while the S/G ratio increased (Tamasloukht et al. 2011).

In addition to changes in lignin composition, concentrations of *p*-coumaric acid, caffeic acid, ferulic acid and sinapic acid were observed to increase in the transgenic *P. dilatatum* lines. Similar results were observed in *L. perenne* transgenic lines (Tu et al. 2010) as well as in lignin-modified *N. tabacum* plants (Chabannes et al. 2001; Dauwe et al. 2007; Prashant et al. 2011), *P. tremula* × *P. alba* (Leplé et al. 2007) and *A. thaliana* (Goujon et al. 2003; Mir Derikvand et al. 2008). This increase indicates that metabolic intermediates have been re-directed into other pathways such as flavonoid biosynthesis, presumably as a result of reduced flux from coumaroyl-CoA, caffeoyl-CoA, and feruloyl-CoA to H- G-, and S- lignin (Leplé et al. 2007; Tu et al. 2010).

Our results suggest that *PdCCR1* is involved in monolignol formation, most notably the production of G-lignin subunits. NIRS analysis estimated that a moderate reduction in total lignin and an increase in the S/G ratio correlate with an increase in digestibility. Similar results were also observed in the *ZmCCR1* ^−^ *Z. mays* mutant where cell wall digestibility improved by 24–28 % with only a 10 % reduction in lignin content (Tamasloukht et al. 2011).

The production of low-lignin C4 grasses with enhanced digestibility is desirable for the forage industry where they are considered more vigorous alternatives to temperate forage species and may increase milk production in the expanding tropical and warm temperate regions (Li et al. 2008; Seidel et al. 2008; Hisano et al. 2009). Engineering of C4 grasses with enhanced digestibility may also provide additional substrates for the cellulosic biofuel production industry. While further experiments are required to identify additional *PdCCR* gene family members, to investigate their roles and to isolate and characterise other enzymes in the lignin-biosynthesis pathway, it is clear that down-regulation of *CCR1* is an effective strategy for the production of C4 forage grasses with decreased lignin content. Future studies will remove the selectable marker cassette by molecular breeding and assess the growth, fertility and agronomic performance of *P. dilatatum* germplasm with enhanced digestibility due to modification of lignin biosynthesis under field conditions.

## Electronic supplementary material

Below is the link to the electronic supplementary material.

Supplementary material 1 (DOC 479 kb)
