# Supplementary material for: Reduced lignin content and altered lignin composition in the warm season forage grass Paspalum dilatatum by down-regulation of a Cinnamoyl CoA Reductase Gene
Source: Transgenic Res. 2014 Feb 7;23(3):503–17. doi: 10.1007/s11248-014-9784-1 (PMC4010725; doi:10.1007/s11248-014-9784-1)
Supplement: Supplementary file 1 — Supplementary material 1 (DOC 479 kb) [file 11248_2014_9784_MOESM1_ESM.doc]

**ELECTRONIC SUPPLEMENTARY MATERIAL**

**Online Resource 1**

Table of oligonucleotide primers and probe sequences used in this study. qPCR: quantitative (real time) polymerase chain reaction. qRT-PCR: quantitative (real-time) reverse transcriptase polymerase chain reaction

|  | **Forward Primer (5' – 3')** | **Reverse Primer (5'-3')** |
| --- | --- | --- |
| Primers designed to the consensus sequence of *CCR1* genes from *Sorghum bicolor*, *Zea mays*, *Panicum virgatum*, *Lolium perenne* and *Phylostachys edulis* | ACCCAGATGACCCGAAGA | TCTTCGGGTCATCTGGGTTC |
|  | GACTACGACGCCATCTGC | TCTTCGGGTCATCTGGGTTC |
|  | CGGGTGGTGTTCACGTC | CGGCTCCACCATTTGCTC |
|  | CTGGTACTGCTACGGCAAGG | CCTTGCCGTAGCAGTACCAG |
|  | GGTGAACGCCAGCATCG | CGATGCTGGCGTTCACC |
|  | GCATCCTCGCCAAGCTCT | AAGAGCTTGGCGAGGATGC |
| Primers to generate a Southern probe specific to *PdCCR1* | TCGGAAGAATCCTACCAAACC | TTCACAGTGTACCCCTTCTCG |
| Primers to generate a Southern probe specific to the promoter of the *Polyubiquitin* gene from *Z.mays* | GGACACCAACCAGCGAACC | GAGCGGCGTACCTTGAAGC |
| Primers to generate a Southern probe specific to *nptII* | CTCTACGGCTACAAGTGG | CATCTCCTTCCAGACCTG |
| Primers for qRT-PCR analysis of *PdCCR* | GACGATCCCGAGCAAATGG | GTGGTGTTCACGTCCTCCA |
| Primers for qRT-PCR analysis of *PdEF1α* | TGATATCGCCCTGTGGAAGT | CAGGGGCATCAATGACAGT |
| Primers for qRT-PCR analysis of *PdCCR* (spanning stop-codon) | CACCTGCCGGTGCCGGTGCCC | CAGATAGATGCCACGCAGTC |
| Primers for detection of *PdEF1α* in genomic DNA by qPCR | GTGGTTATGTGGCCTCGAACTC | TGTGGGAGGTGTGGCAGTC |
| Probe for detection of *PdEF1α* in genomic DNA by qPCR | CGTAGCCGTTGCCGATCTGCCCAG | |
| Primers for detection of *nptII* in genomic DNA by qPCR | GAAGAACTCGTCGAGCATCA | CAGAAGTACGGCATCGACAA |
| Probe for detection of *nptII* in genomic DNA by qPCR | Roche Universal Probe #154 | |
| Primers for detection of the promoter of the *Polyubiquitin* gene from *Z.mays* in genomic DNA by qPCR | GACACCAACCAGCGAACC | ACGGTGGAGCGGAACTCT |
| Probe for detection of the promoter of the *Polyubiquitin* gene from *Z.mays* in genomic DNA by qPCR | Roche Universal Probe #34 | |

**Online Resource 2** Southern hybridisation analysis of genomic DNA (10 µg per lane) isolated from wild-type (WT) and transgenic lines (76, 78 and 87) of *P. dilatatum* digested with *Eco*RI and probed with (A) DIG-labelled fragment of *npt*II and (B) DIG-labelled fragment of the promoter of the polyubiquitin gene 1from *Z. mays* M: DIG Marker III

**Online Resource 3** Levels of hydroxycinnamates and phenylalanine in leaf blade tissue from three transgenic lines. Bar charts show the relative response of the spectral peak of each metabolite. Table shows the means fold change in comparison to wild-type lines (n=3). Asterisks indicate a significant difference relative to the wild-type control (p<0.05)

|  | **78** | | **87** | | **76** | |
| --- | --- | --- | --- | --- | --- | --- |
| **Metabolite** | **Fold change** | ***t* Test**  **(p value)** | **Fold change** | ***t* Test**  **(p value)** | **Fold change** | ***t* Test**  **(p value)** |
| Phenylalanine | 1.1 (up) | 0.354 | 1.5 (up) | 0.072 | 1.4 (up) | 0.121 |
| *p*-coumaric acid | 1.9 (up)* | 0.027 | 1.3 (up)* | 0.001 | 1.8 (up)* | 0.028 |
| Caffeic acid | 1.9 (up)* | 0.005 | 1.6 (up) | 0.081 | 1.3 (up) | 0.221 |
| Ferulic acid | 2.3 (up)* | 0.005 | 1.4 (up)* | 0.001 | 1.9 (up) | 0.074 |
| Sinapic acid | 1.9 (up) | 0.054 | 1.3 (down) | 0.164 | 3.3 (up)* | 0.049 |

**Online Resource 4** (A) Estimate of *in vivo* dry matter digestibility (IVVDMD, %) by near-infrared spectroscopy (NIRS) of *P. dilatatum* leaf blades at vegetative stage from wild-type (WT) and transgenic (87, 76 and 78) lines*.* Error bars indicate standard error (n=3). Asterisks indicate a significant difference (t-test) relative to WT (p<0.05). (B) Scatterplot and linear regression comparing lignin content percentage of cell wall residue (%CWR) to the NIRS estimate of IVVDMD in three replicates of wild-type (black squares) and transgenic events (grey squares). A Pearson correlation coefficient (-0.874), p-value(0.0002) was calculated.

**A**

**B**
